# A novel statistical method for rare-variant association studies in general pedigrees

**DOI:** 10.1186/s12919-016-0029-6

**Published:** 2016-10-18

**Authors:** Huanhuan Zhu, Zhenchuan Wang, Xuexia Wang, Qiuying Sha

**Affiliations:** 1 Department of Mathematical Sciences, Michigan Technological University, 1400 Townsend Drive, Houghton, MI 49931 USA; 2 Department of Mathematics, University of North Texas, 1155 Union Circle #311430, Denton, TX 76203-5017 USA

## Abstract

Both population-based and family-based designs are commonly used in genetic association studies to identify rare variants that underlie complex diseases. For any type of study design, the statistical power will be improved if rare variants can be enriched in the samples. Family-based designs, with ascertainment based on phenotype, may enrich the sample for causal rare variants and thus can be more powerful than population-based designs. Therefore, it is important to develop family-based statistical methods that can account for ascertainment. In this paper, we develop a novel statistical method for rare-variant association studies in general pedigrees for quantitative traits. This method uses a retrospective view that treats the traits as fixed and the genotypes as random, which allows us to account for complex and undefined ascertainment of families. We then apply the newly developed method to the Genetic Analysis Workshop 19 data set and compare the power of the new method with two other methods for general pedigrees. The results show that the newly proposed method increases power in most of the cases we consider, more than the other two methods.

## Background

There is increasing interest in detecting associations between rare variants and complex traits. Although statistical methods to detect common variant associations are well developed, these variant-by-variant methods may not be optimal for detecting associations with rare variants as a result of allelic heterogeneity as well as the extreme rarity of individual variants [1]. Recently, several statistical methods for detecting associations of rare variants were developed for population-based designs, including the cohort allelic sums test [2], the combined multivariate and collapsing method [1], the weighted sum statistic [3], the variable minor allele frequency threshold method [4], the adaptive sum test [5], the step-up method [6], the sequence kernel association test [7], and the test for optimally weighted combination of variants [8].

Meanwhile, quite a few statistical methods for rare-variant association studies have been developed for family-based designs. For any type of study design, the statistical power will be improved if rare variants can be enriched in the samples. If one parent has a copy of a rare allele, half of the offspring are expected to carry it, and, hence, variants that are rare in the general population could be very common in certain families [9]. Therefore, family-based designs may play an important role in rare-variant association studies. Because of the importance of family-based designs in rare-variant association studies, several family-based rare-variant association methods for quantitative traits [10–12] and for qualitative traits [13–15] have been developed. However, most of these methods were developed under the assumption of random ascertainment and family-based designs with random ascertainment may not yield enrichment of rare variants. To analyze the sequencing data in general pedigrees provided by Genetic Analysis Workshop 19 (GAW19), we proposed a novel method to test rare-variant association in general pedigrees for quantitative traits. Applying the proposed method to the GAW19 data set, we compared the power of the proposed method with that of two popular methods for family-based designs.

## Methods

Consider a sample of *n* pedigrees with *n*_*i*_ members in the *i* ^*th*^ pedigree and a genomic region with *M* variants. Let *y*_*ij*_ and *g*_*ij*_ = (*g*_*ij*1_, …, *g*_*ijM*_)^*T*^ denote the trait value and genotypes of the *M* variants in the genomic region for the *j* ^*th*^ individual in the *i* ^*th*^ pedigree. Let *x*_*ij*_ = ∑ _*m* = 1_^*M*^ *w*_*m*_ *g*_*ijm*_ denote the weighted combination of genotypes at the *M* variants, where *w* = (*w*_1_, … ,*w*_*M*_)^*T*^ is a weight function.

For given genotypes, we assume that *y*_*ij*_ ∼ *N*(*a* + *x*_*ij*_ *β*, *σ* ^2^). Using the notation ${g}_i={\left({g}_{i1},\dots, {g}_{i{n}_i}\right)}^T$, the retrospective likelihood is given by

$$ RL={\displaystyle \prod_{i=1}^n \Pr \left({g}_i\left|{y}_{i1},\dots, {y}_{i{n}_i}\right.\right)}={\displaystyle \prod_{i=1}^n\frac{ \Pr \left({y}_{i1},\dots, {y}_{i{n}_i}\left|{g}_i\right.\right) \Pr \left({g}_i\right)}{{\displaystyle {\sum}_{g_i^{*}} \Pr \left({y}_{i1},\dots, {y}_{i{n}_i}\left|{g}_i^{*}\right.\right)} \Pr \left({g}_i^{*}\right)}}={\displaystyle \prod_{i=1}^n\frac{ \exp \left(-{\displaystyle {\sum}_{j=1}^{n_i}}{\left({y}_{ij}-a-{x}_{ij}\beta \right)}^2/2{\sigma}^2\right) \Pr \left({g}_i\right)}{{\displaystyle {\sum}_{g_i^{*}} \exp \left(-{\displaystyle {\sum}_{j=1}^{n_i}}{\left({y}_{ij}-a-{x}_{ij}^{*}\beta \right)}^2/2{\sigma}^2\right)} \Pr \left({g}_i^{*}\right)}}, $$

where ${\displaystyle {\sum}_{g_i^{*}}}$ represents the summation of all possible genotypes. Based on *RL*, the score test statistic for testing the null hypothesis *H*_0_ : *β* = 0 is given by

1 $$ {T}_{score}={U}^2/V $$

where$U={\displaystyle {\sum}_{i=1}^n{\displaystyle {\sum}_{j=1}^{n_i}}}\left({x}_{ij}-\overline{x}\right)\left({y}_{ij}-\overline{y}\right)$, *V* = *w* ^*T*^ *Σw*∑ ^*n*^_*i* = 1_ *y*_*i*_^*T*^ *Φ*_*i*_ *y*_*i*_, ${y}_i={\left({y}_{i1},\dots, {y}_{i{n}_i}\right)}^T$, $\overline{y}=\frac{1}{{\displaystyle {\sum}_{i=1}^n{n}_i}}{\displaystyle {\sum}_{i=1}^n}{\displaystyle \kern.8em {\sum}_{j=1}^{n_i}{y}_{ij}}$, *Φ*_*i*_ is twice the kinship coefficient of the *i* ^*th*^ pedigree, and *Σ* = cov(*g*_11_, *g*_11_) is the covariance matrix of the multiple variant genotype of one individual. *Σ* can be estimated by $\widehat{\varSigma}=\frac{1}{{\displaystyle {\sum}_{i=1}^n{n}_i}}{\displaystyle {\sum}_{i=1}^n{\displaystyle {\sum}_{j=1}^{n_i}}}\left({g}_{ij}-\overline{g}\right){\left({g}_{ij}-\overline{g}\right)}^T$, where $\overline{g}=\frac{1}{{\displaystyle {\sum}_{i=1}^n}{n}_i}{\displaystyle {\sum}_{i=1}^n}{\displaystyle {\sum}_{j=1}^{n_i}{g}_{ij}}$. It is worth pointing out that *T*_*score*_ is equivalent to the quantitative version of the retrospective likelihood score statistic proposed by Schaid et al [16].

Because rare variants are essentially independent, following Pan [17] and Sha et al [8], we replace $\widehat{\varSigma}$ by ${\widehat{\varSigma}}_0= diag\left(\widehat{\varSigma}\right)$. Then, the score test statistic *T*_*score*_ becomes

$$ {T}_0(w)={w}^Tu{u}^Tw/\left({w}^T{\widehat{\varSigma}}_0w{\displaystyle {\sum}_{i=1}^n}{y}_i^T{\varPhi}_i{y}_i\right), $$

where $u={\displaystyle {\sum}_{i=1}^n}{\displaystyle {\sum}_{j=1}^{n_i}}\left({g}_{ij}-\overline{g}\right)\left({y}_{ij}-\overline{y}\right)$. As a function of *w*, *T*_0_(*w*) reaches its maximum when $w={\widehat{\varSigma}}_0^{-1}u$ and the maximum value of *T*_0_(*w*) is ${u}^T{\widehat{\varSigma}}_0^{-1}u/{\displaystyle \sum_{i=1}^n{y}_i^T{\varPhi}_i{y}_i}$. We define the statistic of optimally weighted score test (OW-score) as

$$ {T}_{OW- score}={u}^T{\widehat{\varSigma}}_0^{-1}u/{\displaystyle {\sum}_{i=1}^n{y}_i^T{\varPhi}_i{y}_i={\displaystyle {\sum}_{m=1}^M}\left({u}_m^2/{\sigma}_{mm}\right)/\left({\displaystyle {\sum}_{i=1}^n}{y}_i^T{\varPhi}_i{y}_i\right),} $$

where *σ*_*mm*_ is the (m, m)^*th*^ element of ${\widehat{\varSigma}}_0$ and *u*_*m*_ is the *m* ^*th*^ element of *u*. Under the null hypothesis, *T*_*OW-score*_ is asymptotically distributed as a mixture of independent *χ* ^*2*^ statistics [18, 19]. Alternatively, the distribution of *T*_*OW-score*_ can be approximated by a Satterwaite approximation for the distribution of quadratic forms [7, 20, 21] or a scaled *χ* ^*2*^ distribution [16]. We propose to approximate the distribution of *T*_*OW-score*_ by a scaled *χ* ^*2*^ distribution with the scale *δ* and degrees of freedom *d* estimated by the expectation and variance of *T*_*OW-score*_. Note that *u* ∼ *N*(0, *Σ*∑ _*i* = 1_^*n*^ *y*_*i*_^*T*^ *Φ*_*i*_ *y*_*i*_). We have ${\widehat{\mu}}_T=\widehat{E}\left({T}_{OW- score}\right)= trace\left(\widehat{\varSigma}{\widehat{\varSigma}}_0^{-1}\right)$ and ${\widehat{\sigma}}_T^2=\operatorname{va}\widehat{r}\left({T}_{OW- score}\right)=2 trace\left(\widehat{\varSigma}{\widehat{\varSigma}}_0^{-1}\widehat{\varSigma}{\widehat{\varSigma}}_0^{-1}\right)$. Then, the scale *δ* is estimated as $\widehat{\delta}={\widehat{\sigma}}_T^2/\left(2{\widehat{\mu}}_T\right)$ and the degree of freedom *d* is estimated as $\widehat{d}=2{\widehat{\mu}}_T^2/{\widehat{\sigma}}_T^2$

We compare the performance of our OW-score with (a) WS-score, the score test given by equation (1) with weight given by Madsen and Browning [3] and (b) famSKAT, family-based sequence kernel association test given by Chen et al [11].

## Results

We applied our proposed method as well as the WS-score test and famSKAT to the simulated data from GAW19. All tests were conducted on 849 individuals, from 20 pedigrees, that had no missing genotypes or phenotypes. Sex, age, blood pressure medication status, and smoking status were considered as covariates in this study. We were aware of the underlying simulation model.

There are two related phenotypes, systolic blood pressure (SBP) and diastolic blood pressure (DBP), at three time points. We considered the average of DBP at three time points as the phenotype of interest in our analysis. We compared the power of the three tests (OW-score, WS-score, and famSKAT) to detect association between each of the top 14 genes that influence the phenotype of interest. We used the variants between the first functional single nucleotide polymorphism (SNP) and the last functional SNP in each gene in our analysis. We did not consider *CABP2* because the power of the three tests are essentially the same due to only one variant in this gene. To adjust the effects of the covariates on the phenotype of interest, we first applied a linear model by regressing the phenotype of interest on the covariates: sex, the average of age, the average of blood pressure medication status, and the average of smoking status. The power comparisons based on the 200 replicated data sets are given in Table 1. Significance level is assessed at 5 %. This table shows that the OW-score test identified three genes with power greater than 40 %, famSKAT identified 1 gene with power greater than 40 %, and the WS-score test could not identify any genes with power greater than 40 %. OW-score and famSKAT have different power mainly because they use different weights. Let *w*_*m*_ and *W*_*m*_ denote the weights, rescaled to the interval (0, 1), of the OW-score test and famSKAT for the *m* ^*th*^ variant. Then, *w*_*m*_ > *W*_*m*_ when minor allele frequency (MAF) is less than 0.01; *w*_*m*_ ≤ *W*_*m*_ when MAF is in the interval (0.01, 0.05); *w*_*m*_ > *W*_*m*_ when MAF is greater than 0.05. The OW-score test has much higher power than famSKAT for *RAI1* and *REPIN1* because none of the MAFs of the causal variants in *RAI1* and *REPIN1* are in the interval (0.01, 0.05).

**Table 1:** Power comparisons of the 3 tests using the average of DBP at 3 time points as phenotypes (significance level is assessed at 5 %)

Genes	*T*_*OW-score*_	*T*_*WS-score*_	FamSKAT
***CGN***	0.135	0	0.035
***FLT3***	0.005	0	0.08
***LEPR***	0.05	0.015	0.065
***MAP4***	0.175	0.185	**0.425**
***MTRR***	**0.465**	0.005	0.06
***NRF1***	0	0.005	0.035
***PTTG1IP***	0.02	0.145	0.06
***RAI1***	**0.845**	0.005	0.155
***REPIN1***	**0.915**	0.05	0.085
***SLC35E2***	0.005	0	0.05
***TNN***	0	0	0.035
***ZFP37***	0	0.005	0.005
***ZNF443***	0.01	0.015	0.195
***ZNF544***	0.005	0.015	0.06

Notes: the powers greater than 40 % are in bold

We also evaluated the type I error rate of the proposed OW-score test. To evaluate the type I error, we used 1000 blocks (100 variants in each block) from chromosome 5 that are far from causal variants. In each block, we applied the OW-score test to each of the 200 replicates to test association between genotypes and the phenotype of interest. We obtained 1 *p* value for each replicate and each block. The type I errors of the proposed test were 0.04887, 0.00921, and 0.00131 at significance levels of 0.05, 0.01, and 0.001, respectively. We also considered the average of SBP at three time points as the phenotype of interest, which yielded similar results.

## Discussion

Next-generation sequencing technologies make directly testing rare variant association possible. However, the development of powerful statistical methods for rare-variant association studies is still underway. In this article, we proposed a novel statistical method for rare-variant association studies based on general pedigrees for quantitative traits. The application to the GAW19 data set showed that the proposed method has correct type I error rate and is more powerful than the other two methods against which our method was compared.

We described our method for quantitative traits. For qualitative traits, we can derive a score test similar to that given by equation (1). However, the performance of the proposed method for qualitative traits requires further investigation. Like many statistical methods for rare variant association studies, the proposed method can consider phenotype measurement at only one time point. Statistical methods based on sequence data have been developed for unrelated individuals that have phenotype measurements at multiple time points [22]. From a statistical standpoint, modeling using longitudinal phenotypes is more informative than that using phenotypes at a single time point and thus can increase the power of an association test [22, 23]. Our future work includes extension of the proposed method to longitudinal phenotypes.

## Conclusions

In this article, we developed a novel statistical method for rare variant association studies in general pedigrees (randomly ascertained pedigrees or ascertained pedigrees). Application to the GAW19 data set showed that the newly proposed method is more powerful than the other two methods in most of the cases. Our new method uses a retrospective view, which allows us to account for complex and undefined ascertainment of families. The GAW19 data is based on randomly ascertained pedigrees. Results of applying our method to GAW19 data showed that the proposed method has correct type I error based on random ascertainment. When random ascertainment is violated and ascertainment is based on trait values, the proposed method is expected to have correct type I error. If pedigrees are ascertained because of extreme trait values, the proposed method is expected to have higher power than methods based on randomly ascertained pedigrees.
